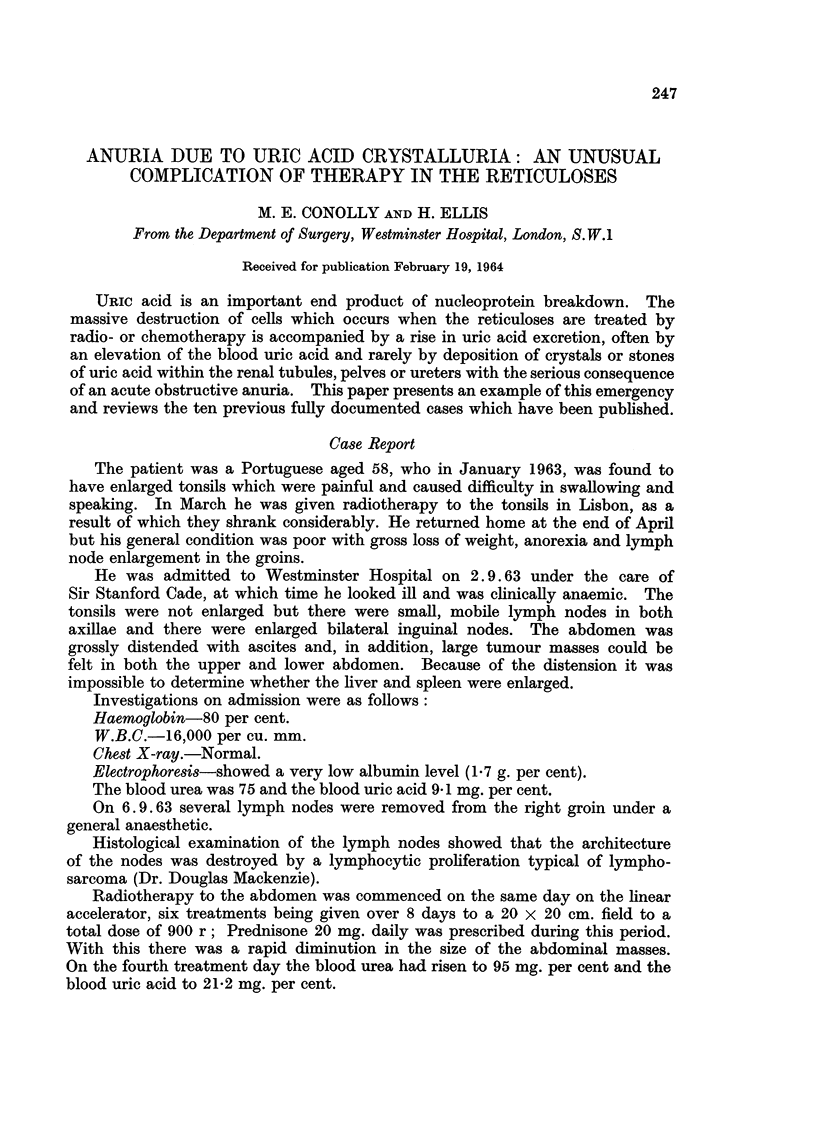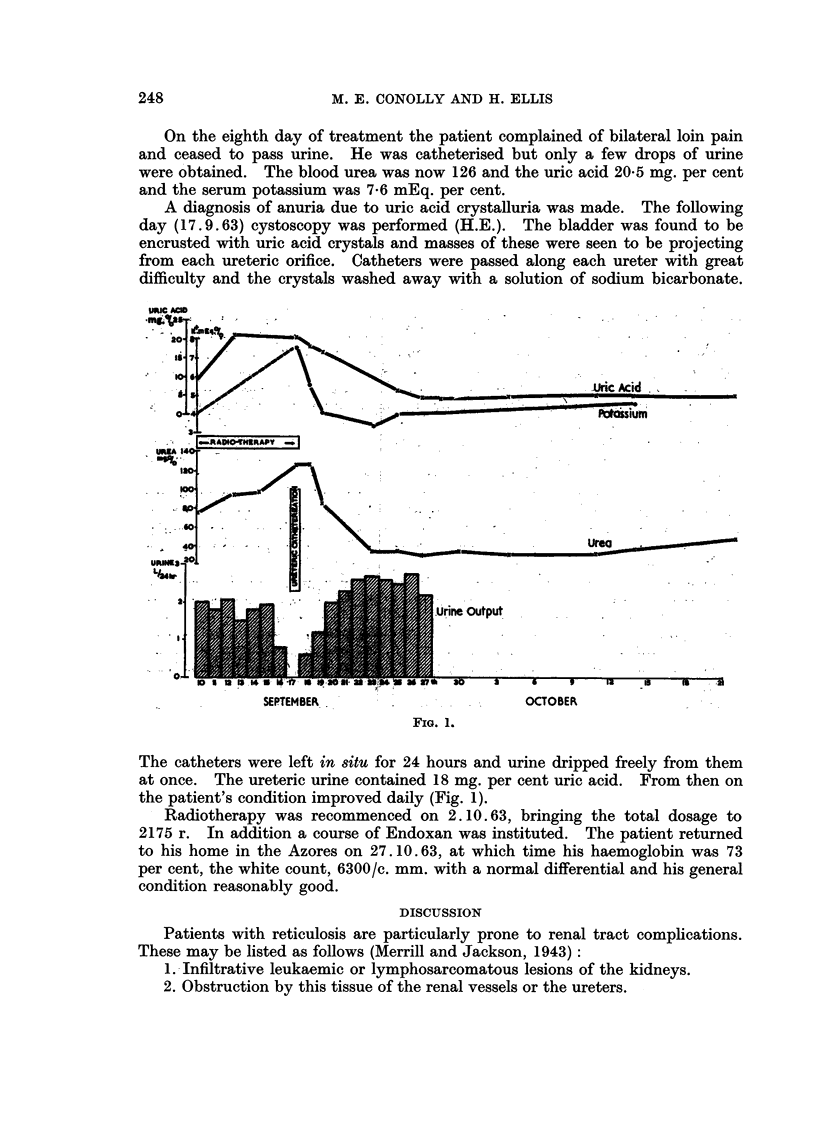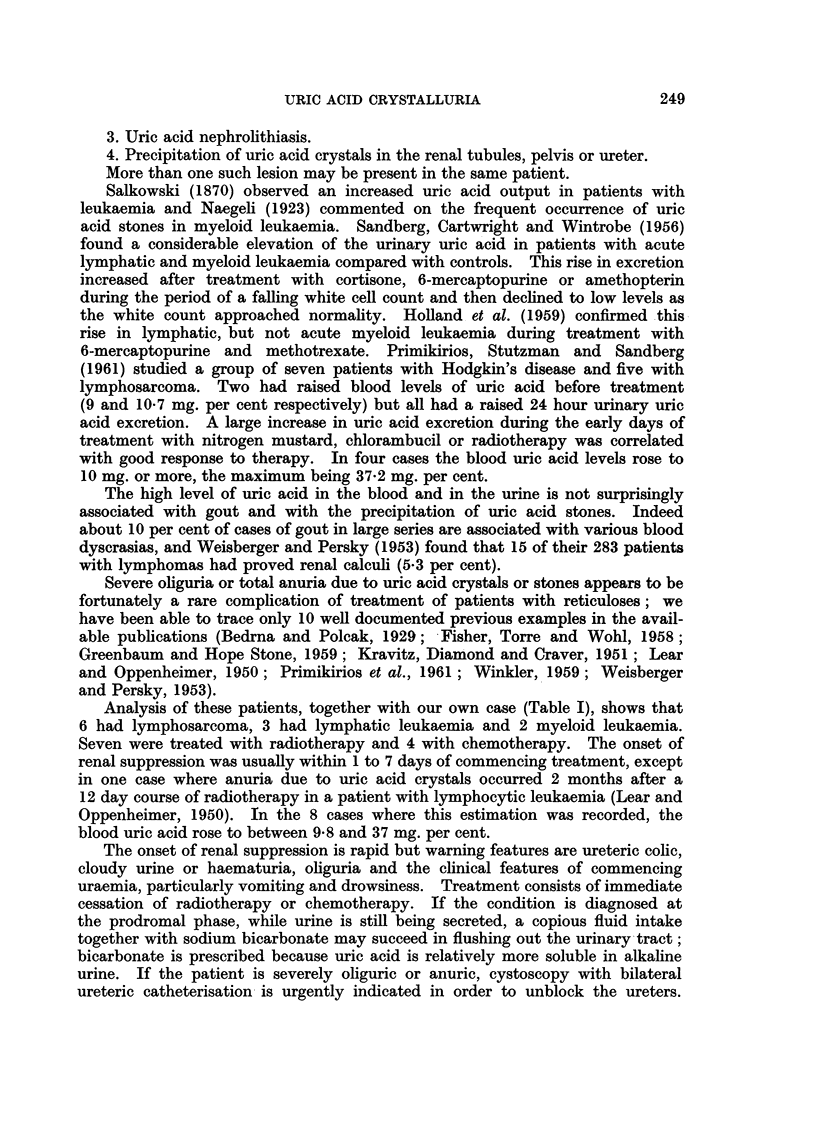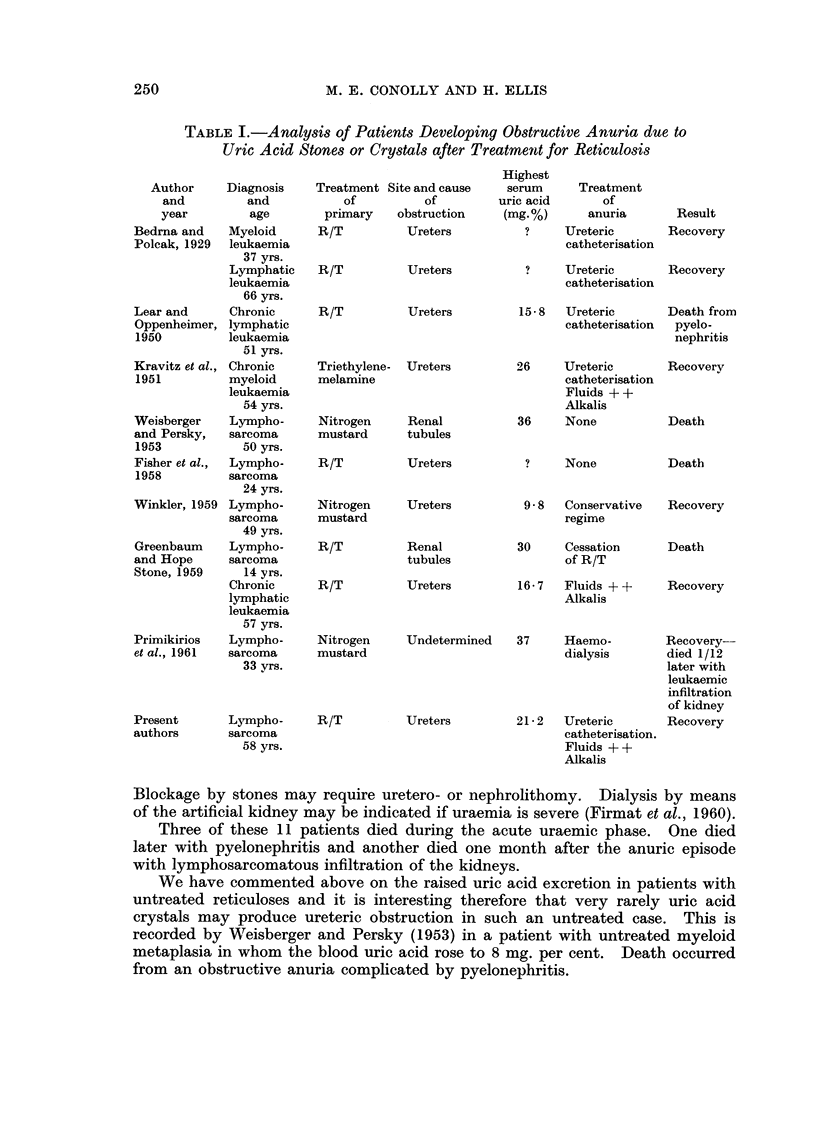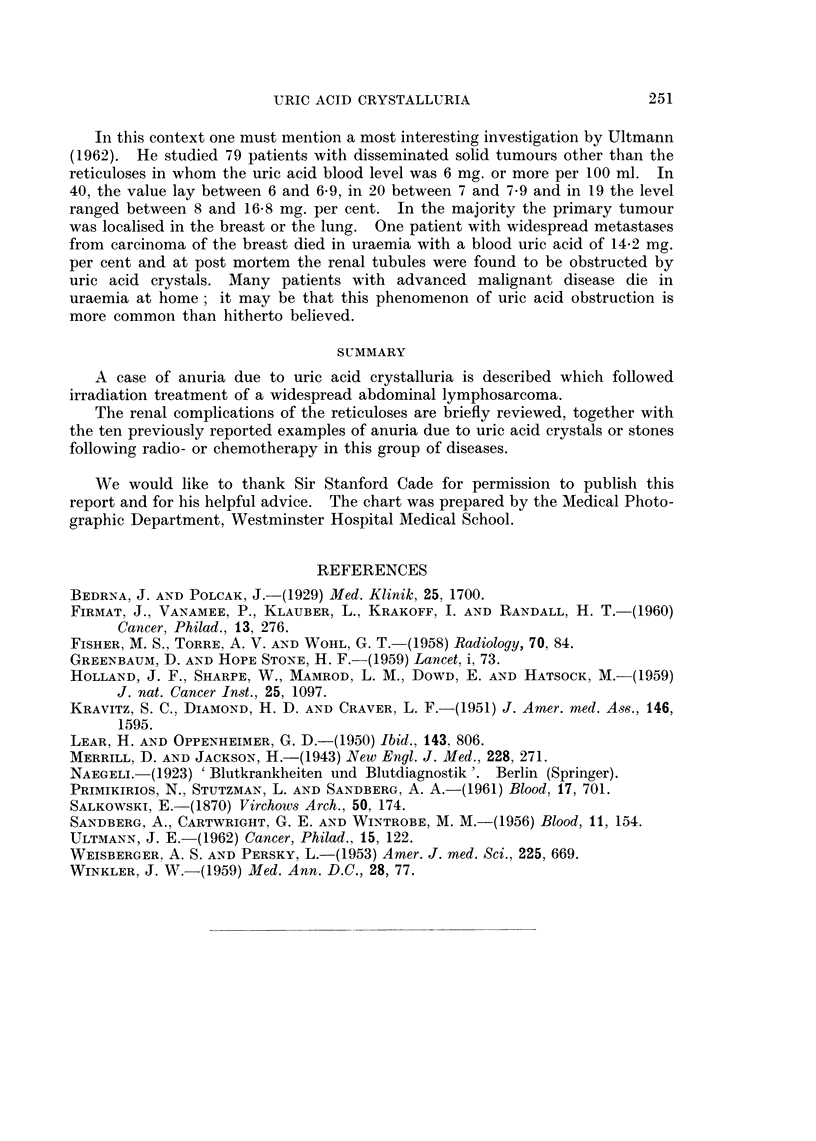# Anuria Due to Uric Acid Crystalluria: An Unusual Complication of Therapy in the Reticuloses

**DOI:** 10.1038/bjc.1964.27

**Published:** 1964-06

**Authors:** M. E. Conolly, H. Ellis


### ANURIA DUE TO URIC ACID CRYSTALLURIA: AN UNUSUAL COMPLICATION OF THERAPY IN THE RETICULOSES

M. E. CONOLLY AND H. ELLIS

*From the Department of Surgery, Westminster Hospital, London, S.W.1*

Received for publication February 19, 1964

URIC acid is an important end product of nucleoprotein breakdown. The massive destruction of cells which occurs when the reticuloses are treated by radio- or chemotherapy is accompanied by a rise in uric acid excretion, often by an elevation of the blood uric acid and rarely by deposition of crystals or stones of uric acid within the renal tubules, pelves or ureters with the serious consequence of an acute obstructive anuria. This paper presents an example of this emergency and reviews the ten previous fully documented cases which have been published.

### *Case Report*

The patient was a Portuguese aged 58, who in January 1963, was found to have enlarged tonsils which were painful and caused difficulty in swallowing and speaking. In March he was given radiotherapy to the tonsils in Lisbon, as a result of which they shrank considerably. He returned home at the end of April but his general condition was poor with gross loss of weight, anorexia and lymph node enlargement in the groins.

He was admitted to Westminster Hospital on 2.9.63 under the care of Sir Stanford Cade, at which time he looked ill and was clinically anaemic. The tonsils were not enlarged but there were small, mobile lymph nodes in both axillae and there were enlarged bilateral inguinal nodes. The abdomen was grossly distended with ascites and, in addition, large tumour masses could be felt in both the upper and lower abdomen. Because of the distension it was impossible to determine whether the liver and spleen were enlarged.

Investigations on admission were as follows:

*Haemoglobin*—80 per cent.

*W.B.C.*—16,000 per cu. mm.

*Chest X-ray*.—Normal.

*Electrophoresis*—showed a very low albumin level (1·7 g. per cent).

The blood urea was 75 and the blood uric acid 9·1 mg. per cent.

On 6.9.63 several lymph nodes were removed from the right groin under a general anaesthetic.

Histological examination of the lymph nodes showed that the architecture of the nodes was destroyed by a lymphocytic proliferation typical of lymphosarcoma (Dr. Douglas Mackenzie).

Radiotherapy to the abdomen was commenced on the same day on the linear accelerator, six treatments being given over 8 days to a 20 × 20 cm. field to a total dose of 900 r; Prednisone 20 mg. daily was prescribed during this period. With this there was a rapid diminution in the size of the abdominal masses. On the fourth treatment day the blood urea had risen to 95 mg. per cent and the blood uric acid to 21·2 mg. per cent.

On the eighth day of treatment the patient complained of bilateral loin pain and ceased to pass urine. He was catheterised but only a few drops of urine were obtained. The blood urea was now 126 and the uric acid 20·5 mg. per cent and the serum potassium was 7·6 mEq. per cent.

A diagnosis of anuria due to uric acid crystalluria was made. The following day (17.9.63) cystoscopy was performed (H.E.). The bladder was found to be encrusted with uric acid crystals and masses of these were seen to be projecting from each ureteric orifice. Catheters were passed along each ureter with great difficulty and the crystals washed away with a solution of sodium bicarbonate.

**Fig. 1.**

The catheters were left *in situ* for 24 hours and urine dripped freely from them at once. The ureteric urine contained 18 mg. per cent uric acid. From then on the patient's condition improved daily (Fig. 1).

Radiotherapy was recommenced on 2.10.63, bringing the total dosage to 2175 r. In addition a course of Endoxan was instituted. The patient returned to his home in the Azores on 27.10.63, at which time his haemoglobin was 73 per cent, the white count, 6300/c. mm. with a normal differential and his general condition reasonably good.

### DISCUSSION

Patients with reticulosis are particularly prone to renal tract complications. These may be listed as follows (Merrill and Jackson, 1943) :

1. Infiltrative leukaemic or lymphosarcomatous lesions of the kidneys.
2. Obstruction by this tissue of the renal vessels or the ureters.

3. Uric acid nephrolithiasis.
4. Precipitation of uric acid crystals in the renal tubules, pelvis or ureter.

More than one such lesion may be present in the same patient.

Salkowski (1870) observed an increased uric acid output in patients with leukaemia and Naegeli (1923) commented on the frequent occurrence of uric acid stones in myeloid leukaemia. Sandberg, Cartwright and Wintrobe (1956) found a considerable elevation of the urinary uric acid in patients with acute lymphatic and myeloid leukaemia compared with controls. This rise in excretion increased after treatment with cortisone, 6-mercaptopurine or amethopterin during the period of a falling white cell count and then declined to low levels as the white count approached normality. Holland *et al.* (1959) confirmed this rise in lymphatic, but not acute myeloid leukaemia during treatment with 6-mercaptopurine and methotrexate. Primikirios, Stutzman and Sandberg (1961) studied a group of seven patients with Hodgkin's disease and five with lymphosarcoma. Two had raised blood levels of uric acid before treatment (9 and 10·7 mg. per cent respectively) but all had a raised 24 hour urinary uric acid excretion. A large increase in uric acid excretion during the early days of treatment with nitrogen mustard, chlorambucil or radiotherapy was correlated with good response to therapy. In four cases the blood uric acid levels rose to 10 mg. or more, the maximum being 37·2 mg. per cent.

The high level of uric acid in the blood and in the urine is not surprisingly associated with gout and with the precipitation of uric acid stones. Indeed about 10 per cent of cases of gout in large series are associated with various blood dyscrasias, and Weisberger and Persky (1953) found that 15 of their 283 patients with lymphomas had proved renal calculi (5·3 per cent).

Severe oliguria or total anuria due to uric acid crystals or stones appears to be fortunately a rare complication of treatment of patients with reticuloses; we have been able to trace only 10 well documented previous examples in the available publications (Bedrna and Polcak, 1929; Fisher, Torre and Wohl, 1958; Greenbaum and Hope Stone, 1959; Kravitz, Diamond and Craver, 1951; Lear and Oppenheimer, 1950; Primikirios *et al.*, 1961; Winkler, 1959; Weisberger and Persky, 1953).

Analysis of these patients, together with our own case (Table I), shows that 6 had lymphosarcoma, 3 had lymphatic leukaemia and 2 myeloid leukaemia. Seven were treated with radiotherapy and 4 with chemotherapy. The onset of renal suppression was usually within 1 to 7 days of commencing treatment, except in one case where anuria due to uric acid crystals occurred 2 months after a 12 day course of radiotherapy in a patient with lymphocytic leukaemia (Lear and Oppenheimer, 1950). In the 8 cases where this estimation was recorded, the blood uric acid rose to between 9·8 and 37 mg. per cent.

The onset of renal suppression is rapid but warning features are ureteric colic, cloudy urine or haematuria, oliguria and the clinical features of commencing uraemia, particularly vomiting and drowsiness. Treatment consists of immediate cessation of radiotherapy or chemotherapy. If the condition is diagnosed at the prodromal phase, while urine is still being secreted, a copious fluid intake together with sodium bicarbonate may succeed in flushing out the urinary tract; bicarbonate is prescribed because uric acid is relatively more soluble in alkaline urine. If the patient is severely oliguric or anuric, cystoscopy with bilateral ureteric catheterisation is urgently indicated in order to unblock the ureters.

TABLE I.—*Analysis of Patients Developing Obstructive Anuria due to Uric Acid Stones or Crystals after Treatment for Reticulosis*

| Author and year | Diagnosis and age | Treatment of primary | Site and cause of obstruction | Highest serum uric acid (mg.%) | Treatment of anuria | Result |
|---|---|---|---|---|---|---|
| Bedrna and Polcak, 1929 | Myeloid leukaemia 37 yrs. | R/T | Ureters | ? | Ureteric catheterisation | Recovery |
| | Lymphatic leukaemia 66 yrs. | R/T | Ureters | ? | Ureteric catheterisation | Recovery |
| Lear and Oppenheimer, 1950 | Chronic lymphatic leukaemia 51 yrs. | R/T | Ureters | 15·8 | Ureteric catheterisation | Death from pyelonephritis |
| Kravitz *et al.*, 1951 | Chronic myeloid leukaemia 54 yrs. | Triethylene-melamine | Ureters | 26 | Ureteric catheterisation Fluids ++ Alkalis | Recovery |
| Weisberger and Persky, 1953 | Lympho-sarcoma 50 yrs. | Nitrogen mustard | Renal tubules | 36 | None | Death |
| Fisher *et al.*, 1958 | Lympho-sarcoma 24 yrs. | R/T | Ureters | ? | None | Death |
| Winkler, 1959 | Lympho-sarcoma 49 yrs. | Nitrogen mustard | Ureters | 9·8 | Conservative regime | Recovery |
| Greenbaum and Hope Stone, 1959 | Lympho-sarcoma 14 yrs. | R/T | Renal tubules | 30 | Cessation of R/T | Death |
| | Chronic lymphatic leukaemia 57 yrs. | R/T | Ureters | 16·7 | Fluids ++ Alkalis | Recovery |
| Primikirios *et al.*, 1961 | Lympho-sarcoma 33 yrs. | Nitrogen mustard | Undetermined | 37 | Haemo-dialysis | Recovery—died 1/12 later with leukaemic infiltration of kidney |
| Present authors | Lympho-sarcoma 58 yrs. | R/T | Ureters | 21·2 | Ureteric catheterisation. Fluids ++ Alkalis | Recovery |

Blockage by stones may require uretero- or nephrolithomy. Dialysis by means of the artificial kidney may be indicated if uraemia is severe (Firmat *et al.*, 1960).

Three of these 11 patients died during the acute uraemic phase. One died later with pyelonephritis and another died one month after the anuric episode with lymphosarcomatous infiltration of the kidneys.

We have commented above on the raised uric acid excretion in patients with untreated reticuloses and it is interesting therefore that very rarely uric acid crystals may produce ureteric obstruction in such an untreated case. This is recorded by Weisberger and Persky (1953) in a patient with untreated myeloid metaplasia in whom the blood uric acid rose to 8 mg. per cent. Death occurred from an obstructive anuria complicated by pyelonephritis.

In this context one must mention a most interesting investigation by Ultmann (1962). He studied 79 patients with disseminated solid tumours other than the reticuloses in whom the uric acid blood level was 6 mg. or more per 100 ml. In 40, the value lay between 6 and 6·9, in 20 between 7 and 7·9 and in 19 the level ranged between 8 and 16·8 mg. per cent. In the majority the primary tumour was localised in the breast or the lung. One patient with widespread metastases from carcinoma of the breast died in uraemia with a blood uric acid of 14·2 mg. per cent and at post mortem the renal tubules were found to be obstructed by uric acid crystals. Many patients with advanced malignant disease die in uraemia at home ; it may be that this phenomenon of uric acid obstruction is more common than hitherto believed.

### SUMMARY

A case of anuria due to uric acid crystalluria is described which followed irradiation treatment of a widespread abdominal lymphosarcoma.

The renal complications of the reticuloses are briefly reviewed, together with the ten previously reported examples of anuria due to uric acid crystals or stones following radio- or chemotherapy in this group of diseases.

We would like to thank Sir Stanford Cade for permission to publish this report and for his helpful advice. The chart was prepared by the Medical Photographic Department, Westminster Hospital Medical School.

### REFERENCES

Bedrna, J. and Polcak, J.—(1929) *Med. Klinik*, **25**, 1700.
Firmat, J., Vanamee, P., Klauber, L., Krakoff, I. and Randall, H. T.—(1960) *Cancer, Philad.*, **13**, 276.
Fisher, M. S., Torre, A. V. and Wohl, G. T.—(1958) *Radiology*, **70**, 84.
Greenbaum, D. and Hope Stone, H. F.—(1959) *Lancet*, i, 73.
Holland, J. F., Sharpe, W., Mamrod, L. M., Dowd, E. and Hatsock, M.—(1959) *J. nat. Cancer Inst.*, **25**, 1097.
Kravitz, S. C., Diamond, H. D. and Craver, L. F.—(1951) *J. Amer. med. Ass.*, **146**, 1595.
Lear, H. and Oppenheimer, G. D.—(1950) *Ibid.*, **143**, 806.
Merrill, D. and Jackson, H.—(1943) *New Engl. J. Med.*, **228**, 271.
Naegeli.—(1923) ‘ Blutkrankheiten und Blutdiagnostik ’. Berlin (Springer).
Primikirios, N., Stutzman, L. and Sandberg, A. A.—(1961) *Blood*, **17**, 701.
Salkowski, E.—(1870) *Virchows Arch.*, **50**, 174.
Sandberg, A., Cartwright, G. E. and Wintrobe, M. M.—(1956) *Blood*, **11**, 154.
Ultmann, J. E.—(1962) *Cancer, Philad.*, **15**, 122.
Weisberger, A. S. and Persky, L.—(1953) *Amer. J. med. Sci.*, **225**, 669.
Winkler, J. W.—(1959) *Med. Ann. D.C.*, **28**, 77.